# Denominator and Workflow Dependence of Age-Specific Viral Detection in Cerebrospinal Fluid

**DOI:** 10.3390/pathogens15090905

**Published:** 2026-08-27

**Authors:** Ji Yeon Kim, Hyeok-Jin Kwon, Jae-Sik Jeon, Jae Kyung Kim

**Affiliations:** 1 Department of Biomedical Laboratory Science, College of Health Sciences, Dankook University, Cheonan 31116, Republic of Koreadnfgurwls@naver.com (H.-J.K.);; 2 Department of Laboratory Medicine, Ewha Womans University Seoul Hospital, Seoul 07804, Republic of Korea

**Keywords:** cerebrospinal fluid, human herpesvirus 6, varicella-zoster virus, laboratory surveillance, molecular diagnostics, testing workflow, denominator

## Abstract

Longitudinal cerebrospinal fluid PCR archives may show apparent age patterns shaped jointly by underlying disease processes and testing architecture. We examined how apparent viral detection patterns varied with denominator choice, target availability, and reconstructed testing configurations among patients tested at a Korean tertiary-care hospital from 2006 to 2026. Clinical diagnoses and adjudication were unavailable, and workflows were reconstructed from reporting configurations rather than definitive platform identifiers. The archive contained 30,481 target-level results from 5353 patient-date episodes in 4560 patients. We summarized per-target positivity, viral-positive composition, and contextual pooled target-level positivity. Human herpesvirus-6 target-level positivity peaked at 31.9% (272/854) at age 1–4 years and comprised 75.8% (91/120) of infant and 84.0% (272/324) of 1–4-year viral-positive detections. However, HHV-6 was represented in 79.9% and 96.1% of episodes in these groups, whereas enterovirus was represented in only 14.4% and 3.9%. VZV was the most frequently detected adult viral target in the full archive, although rankings varied by period. Apparent age patterns were therefore conditioned by target availability, denominator choice, and evolving workflows and should not be interpreted as clinically adjudicated CNS infection epidemiology.

## 1. Introduction

Meningitis and encephalitis are central nervous system syndromes with heterogeneous infectious etiologies, for which timely etiologic evaluation is clinically important [1,2,3,4]. Neurotropic viral detections in cerebrospinal fluid (CSF) vary with age and season [5,6,7]. Polymerase chain reaction (PCR) assays permit rapid detection of multiple bacterial, viral, and fungal targets from limited CSF volumes [8,9,10,11,12,13,14,15]. Interpretation of positive results is particularly context-dependent for varicella-zoster virus (VZV) and herpes simplex virus types 1 and 2 (HSV-1 and HSV-2) [16,17,18,19,20,21], as well as for human herpesvirus 6 (HHV-6) [22,23,24,25,26,27].

Age is especially relevant to the interpretation of HHV-6. Primary HHV-6 infection occurs mainly during infancy and early childhood [23,28]. However, CSF HHV-6 DNA can also reflect reactivation, DNAemia, inherited chromosomally integrated HHV-6 (iciHHV-6), or incidental detection [22,23,24,29]. VZV, HSV-1, and HSV-2 have different age-related profiles [16,17,18,19,20,21,30]. Accordingly, age-stratified laboratory summaries may be clinically informative for post-analytic assessment, but they cannot by themselves identify etiologically equivalent central nervous system infections or estimate population incidence.

Longitudinal laboratory archives pose an additional challenge because target menus, panel width, ordering practices, repeat testing, assay workflows, and extraction/reporting configurations can change over time [12,14,15]. Conceptually, an observed target-level detection in such an archive is jointly shaped by underlying disease occurrence, the probability of CSF sampling, the probability that a specific target is ordered or included, assay performance, and the reporting/extraction configuration. Per-target positivity compares each pathogen with its tested denominator, whereas viral-positive composition describes the distribution of detected viruses among positive results. Both depend on the individual tested and which targets were available. Contextual pooled target-level positivity is additionally influenced by panel width and ordering structure and therefore should not be interpreted as patient-, specimen-, or episode-level diagnostic yield.

Most CSF studies have focused on syndromic-panel performance, diagnostic accuracy, implementation, or clinical utility [9,10,12,14,15,31,32,33], while sequencing studies have shown that predefined panels may miss unexpected viruses [34]. Two recent *Pathogens* studies provide relevant context. Kakouris et al. described age-related HSV-1, HSV-2, and VZV patterns in clinically evaluated central nervous system infections [35]. Fortelny and Marschall showed that herpesvirus positivity in a diagnostic registry was strongly influenced by the selection of submitted specimens [36]. Neither study examined whether age-specific viral rankings remained consistent when target repertoires, denominator definitions, and PCR workflows changed within the same longitudinal archive.

We analyzed a 2006–2026 CSF molecular laboratory archive using three explicitly denominator-specific measures: per-target positivity, viral-positive composition, and contextual pooled target-level positivity. We evaluated how apparent age-specific patterns changed across calendar periods, reconstructed testing configurations, target availability, alternative age boundaries, and same-day or longitudinal de-duplication. The primary aim was methodological: to distinguish patterns that were consistent across the examined descriptive re-analyses from variation introduced by denominator structure, target availability, and local testing practice, rather than to infer the epidemiology of clinically adjudicated viral central nervous system infection.

## 2. Materials and Methods

### 2.1. Study Design, Setting, Eligibility, and Ethics

This retrospective laboratory cohort used data extracted from the laboratory information system of Dankook University Hospital, a tertiary-care hospital in Cheonan, Republic of Korea, and was reported in accordance with the Strengthening the Reporting of Observational Studies in Epidemiology (STROBE) statement and its extension, the REporting of studies Conducted using Observational Routinely-collected health Data (RECORD) statement [37,38]. Records were considered eligible if they contained an interpretable qualitative CSF molecular target result generated during routine clinical care and sufficient information to calculate patient age at testing. Eligibility was defined at the laboratory-result level and did not require diagnostic codes, chart-adjudicated diagnoses, or clinical case definitions. The cohort therefore represents patients who underwent CSF molecular testing in routine practice rather than confirmed cases of meningitis or encephalitis. Linked data on presenting symptoms, prior HHV-6 infection history, immune status, CSF cell counts and biochemical findings, imaging, treatment, and outcomes were unavailable; clinical adjudication of positive detections was not performed. This study was approved by the Institutional Review Board of Dankook University Hospital (DKUH IRB 2026-05-020; 11 June 2026), which waived the requirement for informed consent for the analysis of retrospectively collected, de-identified data. The analytic dataset was extracted on 12 June 2026.

### 2.2. Study Period, Testing Workflows, and Analytic Dataset

The de-identified analytic dataset contained CSF molecular records spanning 14 January 2006 through 30 March 2026. Archived laboratory extraction files were re-audited, and the source-to-analysis flow was reconstructed. The two archived source extracts contained 31,249 target-level rows (26,853 in the earlier extract ending 31 August 2023 and 4396 in the later extract beginning 3 September 2023). The retained archived extracts contained no rows dated 1–2 September 2023; because no independent source log was available, the re-audit could not determine whether this reflected an absence of testing or noncoverage by the retained extracts on those two dates. Predefined result harmonization retained “Negative,” “Positive,” “Weakly Positive,” and “Weak Positive”; the two weak-positive labels were mapped as positive and “Negative” as negative. The archived materials documented this rule-based mapping; no separate case-by-case manual recoding step was documented or identified in the materials available for re-audit. A total of 768 rows with blank, reference-text, footnote-reference, or non-interpretable single-letter result labels were excluded, leaving 30,481 interpretable target-level records from 5353 patient-date testing episodes in 4560 patients (Supplementary Table S12 and Figure S1). No additional rows were excluded for missing age among these interpretable records. This re-audit reconstructs the archived extraction and harmonization pathway but cannot identify tests that were never included in the archived extraction queries. Only 23 target-level records preceded 16 March 2010, after which record accumulation became sustained in the retained archive. These earliest records were retained in the primary analysis, and their influence was examined in a sensitivity analysis. Patient sex was obtained from the harmonized laboratory records, checked for consistency across records belonging to the same patient, and summarized descriptively overall and within age groups.

The primary unit of analysis was the interpretable pathogen-target result. A patient-date testing episode was defined as all CSF molecular target results recorded for the same pseudonymized patient on the same calendar date. Target-level records were retained for the primary result-level measures, whereas patient-date episodes were used to describe testing architecture and same-day co-detection. Target names and qualitative results were harmonized before analysis, and age was calculated at the date of testing. Same-day patient–pathogen duplicate records were retained in the primary analysis and examined separately. Target-level records were not collapsed across multiplex panels because contextual pooled target-level positivity was intended only as a contextual description of result-level positivity under the local testing structure; it was not interpreted as patient-, specimen-, or episode-level diagnostic yield. Viral-positive composition and per-target positivity used their own explicitly defined denominators.

The institutional testing repertoire evolved during the study period. Before 10 November 2020, clinically selected CSF molecular testing was performed using commercial conventional multiplex PCR kits with electrophoretic detection (Seegene Inc., Seoul, Republic of Korea): the Seeplex Meningitis-V1 ACE Detection kit (Seegene Inc., Seoul, Republic of Korea) for herpesvirus targets (HSV-1, HSV-2, VZV, EBV, CMV, and HHV-6) and the Seeplex Meningitis-B ACE Detection kit Seegene Inc., Seoul, Republic of Korea for bacterial targets (*Escherichia coli* K1, *Haemophilus influenzae*, *Listeria monocytogenes*, *Neisseria meningitidis*, *Streptococcus agalactiae*, and *Streptococcus pneumoniae*). In this study, “selected testing” refers to workflows in which predefined pathogen sets were ordered and reported according to clinical request, rather than to singleplex testing. The local ordering, reporting configurations, and target availability varied over time; hence, the target combinations represented in the retained analytic dataset did not always reproduce complete manufacturer-defined repertoires. Although EBV was included in the documented historical herpesvirus repertoire, the archived pre-September-2023 extraction query explicitly enumerated the retained test codes and did not include an EBV-specific code. EBV target-level records were therefore beyond the scope of that archived extraction and could not be analyzed; historical EBV test volume or positivity cannot be inferred from this dataset. Target-specific retention was also incomplete for CMV: within pre-10 November 2020 selected/non-14-target episodes, CMV was represented in only 1.4–10.7% of episodes across age groups, whereas HSV-1, HSV-2, VZV, and HHV-6 were represented in 74.0–95.3% (Supplementary Table S14). Retention was therefore target-specific rather than uniform, and per-target denominators should be interpreted as retained-and-reported denominators rather than complete records of assays performed. The BioFire FilmArray Meningitis/Encephalitis Panel (ME Panel; bioMérieux, Marcy-l’Étoile, France) became available on 10 November 2020, although selected testing remained in use for some orders.

Throughout, “workflow” denotes the general testing process, whereas “configuration” denotes the operational category reconstructed from reporting fields. The FilmArray panel detects 14 central nervous system pathogen targets: *Escherichia coli* K1, *Haemophilus influenzae*, *Listeria monocytogenes*, *Neisseria meningitidis*, *Streptococcus agalactiae*, *Streptococcus pneumoniae*, cytomegalovirus, enterovirus, HSV-1, HSV-2, HHV-6, human parechovirus, VZV, and *Cryptococcus neoformans*/*gattii* [8,9]. Documented assay characteristics, target repertoires, ordering structures, reported result units, extraction scope, and limits of retrospective reconstruction are summarized in Supplementary Table S10. For configuration-specific summaries, patient-date episodes were classified using the harmonized reporting-configuration field as selected/non-14-target configurations or reconstructed complete 14-target configurations. Each episode classified as a reconstructed complete 14-target configuration was then verified to contain at least one interpretable result for all 14 panel targets after same-day patient–pathogen de-duplication; all such episodes met this verification criterion (Supplementary Table S14). Because reliable platform identifiers were not retained for all records, these categories represent reconstructed reporting configurations rather than definitive assay identities. Quantitative viral-load data were unavailable, target availability varied over time, and no assay-performance comparisons were undertaken.

Calendar-period summaries compared records obtained before 10 November 2020 with those obtained on or after that date, whereas workflow-specific summaries described records within the reconstructed testing configurations without inferential comparison. Calendar period was not used as a proxy for workflow because selected testing continued after panel availability. The operational split also coincided with the COVID-19 pandemic; consequently, calendar-period contrasts may reflect pandemic-related changes in healthcare utilization, referral, pathogen circulation, case mix, and diagnostic behavior in addition to assay availability.

### 2.3. Age Groups and Outcome Definitions

Six age groups were used: <1 year, 1–4 years, 5–17 years, 18–39 years, 40–64 years, and ≥65 years. These categories were chosen to distinguish infancy, early childhood, school-age/adolescence, younger adulthood, middle age, and older adulthood while maintaining usable denominators. Because the adult cut-off points are pragmatic, rather than biologically unique, alternative age-boundary schemes were examined in the sensitivity analysis.

We calculated three complementary measures. Per-target positivity was the number of positive results divided by all interpretable results for the specified target within an age group. The main figure emphasized HHV-6, VZV, HSV-1, and HSV-2 because these were identified descriptively after aggregation as the four most frequently detected herpesviruses, accounting for 988/1010 viral detections (97.8%); this was not a prespecified etiologic target set. Per-target denominators and positivity for all seven viral targets are provided in Supplementary Table S15. Viral-positive composition was the share contributed by each viral target among all viral-positive detections within an age group. Contextual pooled target-level positivity was the proportion of positive target-level results among all interpretable target-level results within an age group and was expected to vary with target availability, panel width, and ordering practice. The three measures were reported separately because they answer different laboratory questions.

### 2.4. Statistical Analysis and Sensitivity Analyses

Analyses were descriptive. Counts and proportions were calculated, and patient-cluster bootstrap intervals were used for the principal age-specific estimates. In each of 10,000 resamples, pseudonymized patients were sampled with replacement and all target-level records for each sampled patient were retained together; multiplicity was preserved when a patient was sampled more than once. Percentile 95% confidence intervals (CIs) were calculated for per-target positivity, viral-positive composition, and contextual pooled target-level positivity. These intervals quantify patient-resampling uncertainty conditional on the observed testing structure; they do not incorporate uncertainty caused by secular changes in target availability, clinician ordering, workflow, referral, or other ascertainment processes. Formal hypothesis testing was not emphasized. Pattern consistency was evaluated descriptively across the predefined sensitivity analyses; no formal equivalence margin or hypothesis test for stability was used. Full software versions, random-seed information, and analysis documentation are provided in the reproducibility package (Supplementary File S2).

Alternative age-boundary schemes were examined to assess whether the main conclusions depended on the prespecified cut points. A separate sensitivity analysis excluded the 23 earliest target-level records, all of which preceded 16 March 2010, and recalculated age-specific contextual pooled target-level positivity and viral-positive composition.

To assess the influence of duplicate rows recorded for the same patient, pathogen, and calendar date, a same-day patient–pathogen de-duplication analysis was performed. Duplicate rows were collapsed to one result. When duplicate same-day results were discordant, the de-duplicated result was classified as positive if any corresponding record was positive.

To characterize testing architecture, same-day patient–pathogen de-duplicated records were grouped into patient-date episodes. For each age group, we calculated the number of unique patients and episodes, median number of distinct viral targets represented per episode, proportion of episodes in which each viral target was represented, and proportion meeting the reconstructed complete 14-target definition. Target availability was further tabulated by age group, calendar period, and reconstructed configuration (Supplementary Tables S13 and S14). For seasonality, months were grouped as winter (December–February), spring (March–May), summer (June–August), and autumn (September–November); descriptive seasonal target-level positivity was reported without inferential testing (Supplementary Table S16). Same-day co-detection was defined as two or more distinct positive pathogen targets within one patient-date episode after patient–pathogen de-duplication and was summarized as viral–viral, viral–nonviral, or nonviral–nonviral molecular co-detection (Supplementary Table S17).

In a separate longitudinal first-positive de-duplication analysis, positive detections were sorted by pseudonymized patient identifier, pathogen, and date, and only the first positive detection for each patient–pathogen combination was retained. Because comparable episode-level target denominators were unavailable across workflows, this analysis was limited to positive-detection composition and did not estimate patient-, specimen-, or episode-level positivity.

### 2.5. Use of Generative AI in Manuscript Preparation

During manuscript preparation, ChatGPT-5.6 (OpenAI, San Francisco, CA, USA;web application, https://chatgpt.com/, accessed 25 August 2026) was used to assist with code generation for the additional descriptive analyses, organization of point-by-point reviewer responses, and English-language editing. Claude (Anthropic; web application, https://claude.ai/, accessed 18 August 2026) was used to assist with a secondary consistency check of the manuscript and revision package. Neither tool supplied source data nor independently adjudicated results. AI-assisted code, checks, and outputs were reviewed by the authors, and aggregate values reported in the manuscript were cross-checked against the analysis-ready data and internal cross-table totals before inclusion. The authors retain responsibility for all analysis rules, numerical outputs, interpretations, and manuscript content.

## 3. Results

The 4560 unique patients contributed 5353 patient-date testing episodes. At the patient-date episode level, the median age at testing was 6.95 years (IQR, 0.55–35.80). Among unique patients, 2608 (57.2%) were male, and 1952 (42.8%) were female. Age-group-specific cohort structure is provided in Supplementary Table S11.

Testing architecture differed materially by age group (Supplementary Tables S13 and S14). The median number of distinct viral targets represented per patient-date episode was four (IQR, 4–4) in each age group, although specific target availability differed. Among infants, HHV-6 was represented in 1213/1518 episodes (79.9%), whereas enterovirus and parechovirus were each represented in 219/1518 (14.4%); 219/1518 infant episodes (14.4%) met the reconstructed complete 14-target definition. Among children aged 1–4 years, HHV-6 was represented in 848/882 episodes (96.1%), whereas enterovirus and parechovirus were each represented in 34/882 (3.9%); only 34/882 episodes (3.9%) met the reconstructed complete 14-target definition. These differences show that the age-specific positive-detection composition was generated under unequal target availability.

### 3.1. Per-Target Positivity: Four Most Frequently Detected Herpesviruses

**Figure 1:**
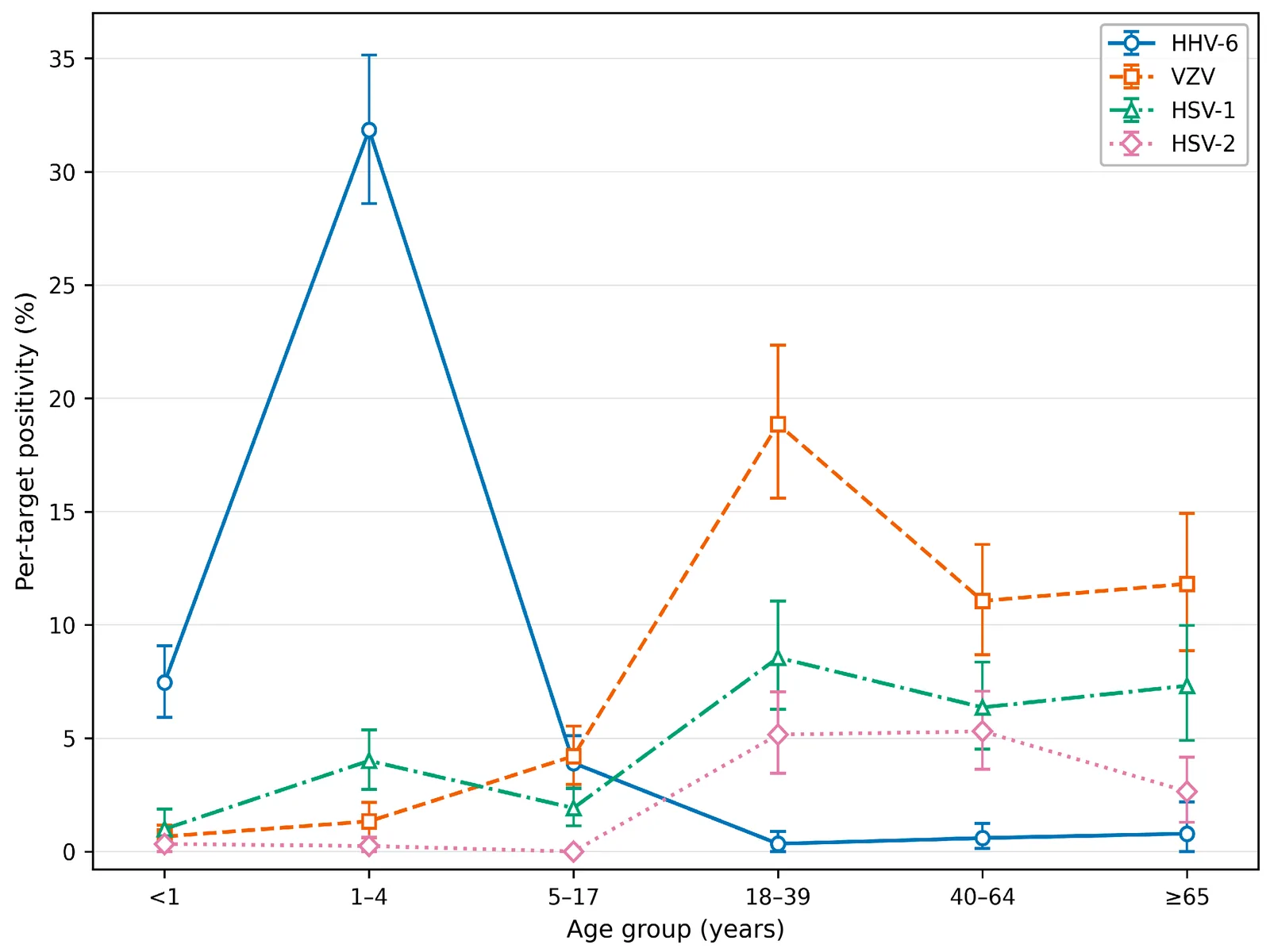
Per-target CSF molecular positivity for HHV-6, VZV, HSV-1, and HSV-2 by age group. Points represent positive results divided by all interpretable results for the specified target within each age group; error bars show 95% confidence intervals obtained from 10,000 patient-cluster bootstrap resamples. Target-specific numerators and denominators are reported in Supplementary Table S3. Corresponding data for all viral targets are reported in Supplementary Table S15. The confidence intervals quantify patient-resampling uncertainty conditional on the observed testing structure and do not incorporate structural uncertainty arising from target availability, ordering, workflow, referral, or other ascertainment changes. Abbreviations: HHV-6, human herpesvirus 6; VZV, varicella-zoster virus; HSV-1, herpes simplex virus type 1; HSV-2, herpes simplex virus type 2.

Of the 1058 positive target-level results, 1010 (95.5%) were viral detections. HHV-6, VZV, HSV-1, and HSV-2 accounted for 988/1010 viral detections (97.8%) and were therefore displayed in the main per-target figure; corresponding denominators and positivity for all viral targets are provided in Supplementary Table S15. HHV-6 target-level positivity peaked at 31.9% (272/854) among children aged 1–4 years. VZV target-level positivity was highest among adults aged 18–39 years (18.9%, 106/562) and was 11.1% (73/660) at 40–64 years and 11.8% (58/491) at ≥65 years. HSV-1 target-level positivity was also highest at 18–39 years (8.6%, 48/561), whereas HSV-2 target-level positivity was 5.2% (29/562) at 18–39 years and 5.3% (35/660) at 40–64 years (Figure 1; Supplementary Tables S3 and S15).

### 3.2. Age-Specific Viral Detection Composition

**Figure 2:**
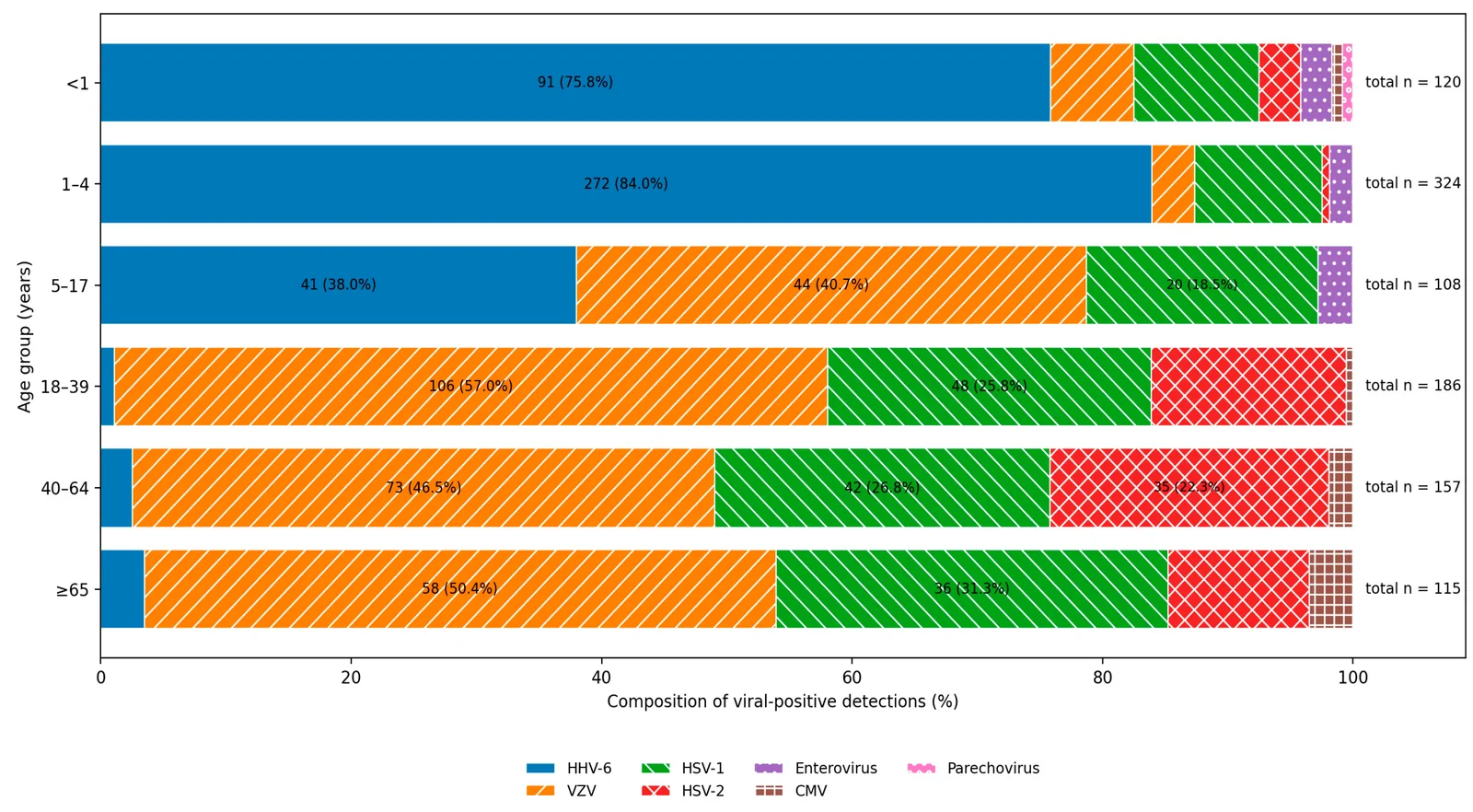
Composition of viral-positive CSF molecular detections by age group. Percentages use the viral-positive denominator within each age stratum and therefore remain conditional on target availability. Where space permits, segments are labeled with the absolute number of detections and the corresponding percentage; the total viral-positive denominator is shown to the right of each bar. These distributions describe laboratory target detections conditional on which targets were represented and should not be interpreted as etiologic fractions of clinically adjudicated CNS infection. Patient-cluster bootstrap 95% confidence intervals are provided in Supplementary Table S2. CSF, cerebrospinal fluid; CMV, cytomegalovirus; HHV-6, human herpesvirus 6; HSV-1, herpes simplex virus type 1; HSV-2, herpes simplex virus type 2; VZV, varicella-zoster virus.

Among the 1010 viral-positive detections, HHV-6 accounted for 91/120 (75.8%) in infants and 272/324 (84.0%) among children aged 1–4 years, under the target availability described above. At 5–17 years, VZV accounted for 44/108 (40.7%) and HHV-6 for 41/108 (38.0%). VZV accounted for the largest share among adults aged 18–39 years (106/186, 57.0%), 40–64 years (73/157, 46.5%), and ≥65 years (58/115, 50.4%). These shares are distributions of laboratory target detections conditional upon which targets were represented, not etiologic fractions (Figure 2; Supplementary Tables S2 and S13).

### 3.3. Contextual Pooled Target-Level Positivity

**Figure 3:**
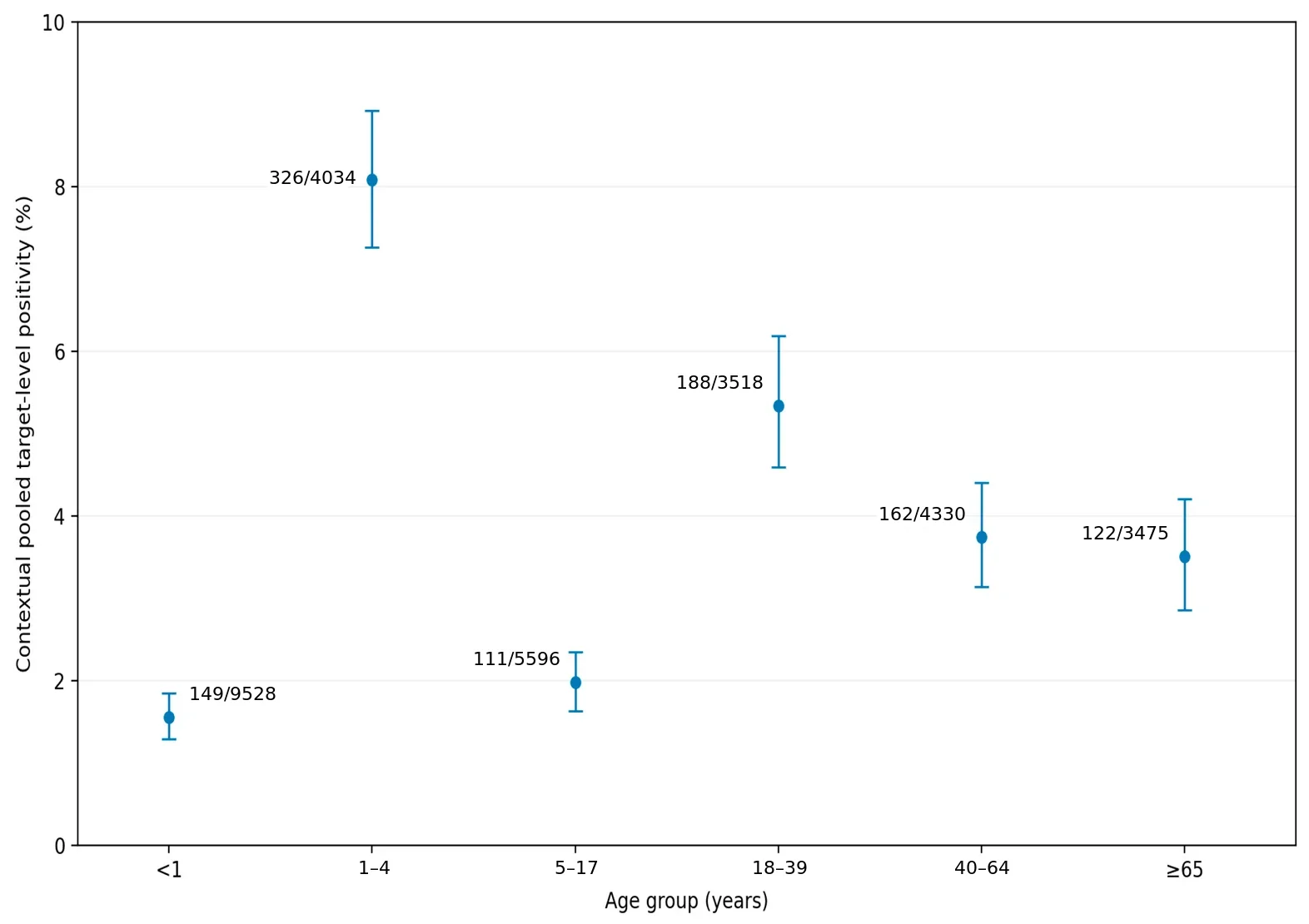
Contextual age-specific pooled target-level CSF molecular positivity with patient-cluster bootstrap 95% confidence intervals. Point estimates are positive target results divided by all interpretable target results within each age group. Positive/total target-level counts are annotated adjacent to each point. This measure is not patient-, specimen-, or episode-level diagnostic yield and is strongly conditioned by panel width, target availability, and ordering structure. The confidence intervals quantify patient-resampling uncertainty conditional on the observed testing structure and do not incorporate structural uncertainty arising from target availability, ordering, workflow, referral, or other ascertainment changes.

Contextual pooled target-level positivity was 1.56% (149/9528) in infants, 8.08% (326/4034) at 1–4 years, 1.98% (111/5596) at 5–17 years, 5.34% (188/3518) at 18–39 years, 3.74% (162/4330) at 40–64 years, and 3.51% (122/3475) at ≥65 years (Figure 3; Supplementary Table S1). Because the number of targets reported per episode differed by age group and calendar period, these values describe the local result-level testing structure and are not patient-, specimen-, or episode-level diagnostic yield.

### 3.4. Calendar-Period and Workflow Context

Calendar-period and reconstructed-configuration dataset structure are summarized in Table 1. Before 10 November 2020, 17,150 target-level results included 846 positives (4.93%); on or after that date, 13,331 target-level results included 212 positives (1.59%). Of the 1578 patient-date episodes in the later period, 1008 (63.9%) remained within selected/non-14-target configurations, and 570 met the reconstructed complete 14-target definition. These are descriptive laboratory contrasts, not causal effects of workflow introduction (Supplementary Tables S7, S8 and S14).

The age group with the highest contextual pooled target-level positivity was 18–39 years before 10 November 2020 (158/1745, 9.05%) and 1–4 years on or after that date (64/1003, 6.38%). HHV-6 accounted for the largest share of viral-positive detections in infants and children aged 1–4 years in both calendar periods. In the 5–17-year group, VZV led before 10 November 2020 (38/83, 45.8%), whereas HHV-6 led on or after that date (15/25, 60.0%). VZV led all adult groups before 10 November 2020; on or after that date, VZV led in the 18–39-year group and HSV-1 at ages 40–64 and ≥65 years (Supplementary Table S8). Thus, the full-archive adult VZV ranking was not invariant across calendar periods.

Workflow-specific pediatric composition was examined separately from calendar-period composition. In selected/non-14-target configurations, HHV-6 accounted for 91/115 (79.1%) infant and 268/313 (85.6%) 1–4-year viral-positive detections. In reconstructed complete 14-target configurations, only five and eleven viral-positive detections were available for these age groups; enterovirus accounted for 3/5 and 6/11 detections, whereas HHV-6 accounted for 0/5 and 4/11, respectively (Supplementary Table S7B). Because the complete-configuration positive denominators were extremely small, these rankings are presented only as exploratory workflow-specific observations and are not used to infer an epidemiological shift.

### 3.5. Additional Descriptive Context

Same-day co-detection was uncommon. Among 1039 patient-date episodes with at least one positive target, 18 contained two or more distinct positive targets after same-day patient–pathogen de-duplication: 17 viral–viral co-detections and one viral–nonviral co-detection. The most frequent combinations were HSV-1 + VZV (*n* = 7), HHV-6 + VZV (*n* = 5), and HHV-6 + HSV-1 (*n* = 4). Among 414 HHV-6-positive patient-date episodes, nine had at least one additional simultaneously detected positive target. These findings describe molecular co-detection and do not establish clinical coinfection (Supplementary Table S17).

Seasonal summaries showed target-specific variation without changing the interpretation that testing architecture was heterogeneous. Enterovirus target-level positivity was 0/139 in winter, 0/104 in spring, 7/156 (4.49%) in summer, and 5/173 (2.89%) in autumn. HHV-6 positivity varied from 7.46% to 9.52% across seasons, whereas VZV varied only from 6.07% to 6.58%. In the retained archive, enterovirus and parechovirus target results occurred only on or after 10 November 2020 within reconstructed complete 14-target configurations, so their apparent seasonal pattern is inseparable from calendar-period and configuration availability. These summaries are descriptive and were not adjusted for calendar period, workflow, or changing target availability (Supplementary Tables S14 and S16).

### 3.6. Sensitivity Analyses

The patient-cluster bootstrap 95% CI for contextual pooled target-level positivity among children aged 1–4 years was 7.26–8.92%; the corresponding CIs for the HHV-6 shares in infants and children aged 1–4 years were 66.1–85.1% and 80.1–87.7%, respectively (Supplementary Tables S1 and S2).

The exact age group with the highest contextual pooled target-level positivity changed under alternative age-boundary schemes. HHV-6, however, remained the leading viral target in the youngest age strata with viral-positive detections across the schemes examined (Supplementary Table S5).

Exclusion of the 23 target-level records obtained before 16 March 2010 did not materially alter age-specific contextual pooled target-level positivity estimates or the leading viral target in any age group (Supplementary Table S9).

Same-day patient–pathogen de-duplication reduced the target-level denominator from 30,481 to 30,334 while leaving the number of positive target results unchanged at 1058 (Supplementary Table S7, part A).

In the separate longitudinal first-positive de-duplication analysis, 1058 positive target results were reduced to 1002 first patient–pathogen positive detections. Forty patient–pathogen combinations had repeat positivity, contributing 56 additional positive rows. Viral positives were reduced from 1010 to 962. After longitudinal first-positive de-duplication, HHV-6 remained the leading detected target in infants (87/111, 78.4%) and among children aged 1–4 years (250/301, 83.1%); the 5–17-year group had equal HHV-6 and VZV shares (41/105 each, 39.0%); and VZV remained the leading detected target in all adult groups (Supplementary Table S6). These results show consistency of the positive-detection ranking under this specific de-duplication rule, not formal statistical robustness.

## 4. Discussion

The central finding of this study is methodological: apparent age-specific viral patterns in a longitudinal CSF molecular archive were strongly conditioned by what was tested and how the denominator was defined. The archive captures only the final laboratory output of these processes and cannot disentangle the individual contributions of disease occurrence, CSF sampling, target ordering, assay performance, and reporting configuration. In the full archive, HHV-6 accounted for the largest share of viral-positive detections in early childhood. This ranking was consistent across several predefined descriptive re-analyses. However, the testing-architecture analysis revealed that HHV-6 was represented in 79.9% of infant and 96.1% of 1–4-year episodes, whereas enterovirus was represented in only 14.4% and 3.9%, respectively. The much smaller reconstructed complete 14-target pediatric subsets produced a different ranking but contained only five and eleven viral-positive detections. Taken together, these results support interpretation of the headline pediatric pattern as a laboratory detection pattern generated under selective and changing target availability, rather than an estimate of the predominant cause of viral central nervous system infection.

VZV accounted for the largest share of adult viral detections in the full archive and after first-positive de-duplication, which is directionally consistent with the results of a recent five-year study from Western Greece [35]. That study incorporated clinical, CSF, and imaging findings and focused on HSV-1, HSV-2, and VZV, whereas the present study addresses a different question: how laboratory rankings change under different denominators and testing contexts. Importantly, the adult ranking was not uniform over time; in the later calendar period, HSV-1 led at ages 40–64 and ≥65 years. Accordingly, VZV was the most frequently detected adult viral target in the full archive, while adult rankings varied by calendar period.

Primary HHV-6 infection usually occurs in infancy or early childhood [23,28]; thus, the age distribution of HHV-6 molecular detections is biologically plausible. Nevertheless, a CSF HHV-6 PCR signal is not etiologically equivalent to detection of every other viral target. CSF HHV-6 DNA may reflect primary infection, reactivation, DNAemia, inherited chromosomally integrated HHV-6, or incidental detection [22,23,29]. This study lacked data for prior HHV-6 infection history, quantitative viral load, paired blood testing, iciHHV-6 assessment, immune status, CSF inflammatory indices, and clinical syndrome adjudication. Accordingly, the HHV-6 results should be interpreted only as molecular detection patterns among tested patients, rather than evidence that HHV-6 was the predominant cause of early-childhood viral CNS disease. A recent evaluation of the BioFire ME panel similarly emphasized the uncertain clinical attribution of many HHV-6 detections [39].

The workflow-specific pediatric comparison should therefore be interpreted cautiously. The reconstructed complete 14-target configuration contained only five viral-positive detections in infants and eleven among children aged 1–4 years, which is insufficient for stable ranking comparisons. Expanded target repertoires can change the observed pathogen distribution [34]; however, these small subgroups cannot confirm or refute the full-archive pediatric pattern. They instead illustrate why target availability and selection-by-testing must be shown alongside positive-detection composition.

### 4.1. Implications for Laboratory Surveillance and Audit

Longitudinal laboratory surveillance can be distorted when target menus and ordering practices change. Contextual pooled target-level positivity fell from 4.93% before 10 November 2020 to 1.59% thereafter; however, almost two-thirds of later patient-date episodes still used selected/non-14-target configurations. The observation unit and denominator must therefore be specified before result-level positivity is compared over time. Per-target positivity describes a pathogen-specific tested denominator, viral-positive composition describes the distribution among detected viruses, and contextual pooled target-level positivity reflects the local mixture of targets and workflows rather than conventional diagnostic yield. The additional target-availability tables make the ascertainment structure explicit and show why denominator-specific measures should not be treated interchangeably. For practical audit of longitudinal LIS archives, age- or time-stratified detection summaries should therefore be accompanied by explicit observation units and target-availability information before changes are attributed to disease epidemiology.

### 4.2. Limitations

This study has several limitations. Clinical information, including symptoms, prior HHV-6 infection history, immune status, CSF cellular and biochemical findings, imaging, treatment, and outcomes, was unavailable; positive detections could therefore not be adjudicated as true central nervous system infections or attributed etiologically. The primary unit was the target-level result, so patients or episodes with wider panels contributed more result-level denominator observations. Patient-date episodes were used to describe testing architecture and co-detection because unique specimen identifiers and fully comparable specimen-level denominators were unavailable.

The archived source extraction files could be re-audited sufficiently to reconstruct the 31,249-to-30,481 result flow and reason-specific exclusions; however, they do not establish completeness for tests that were outside the archived extraction queries. No independent annual-volume, middleware, or instrument-log dataset was available to verify completeness outside the archived extraction queries. In particular, the documented historical herpesvirus repertoire included EBV, whereas the archived pre-September-2023 extraction query did not include an EBV-specific code; historical EBV testing volume and positivity therefore remain unavailable. Retention was also partial for CMV, which appeared in only a small minority of pre-2020 selected episodes despite belonging to the same documented herpesvirus kit repertoire. Target-specific incompleteness is therefore a demonstrated property of this archive, and absolute target-level volumes should not be interpreted as complete institutional testing volumes. Patient-cluster bootstrap intervals account for repeated observations within patients but not for structural uncertainty arising from target availability, ordering, referral, workflow, assay changes, or other ascertainment processes. The 10 November 2020 calendar split coincided with the COVID-19 pandemic and does not permit isolation of workflow effects from pandemic-related changes in healthcare utilization, referral, pathogen circulation, and diagnostic behavior. Reliable platform identifiers were incomplete, so workflow categories are reconstructed reporting configurations rather than definitive assay identities. The pediatric complete-configuration subgroups were very small. Finally, this single-tertiary-center tested population was strongly pediatric (episode-level median age, 6.95 years), likely reflecting local referral and testing practice; the age distribution and target rankings should not be generalized to population-level CNS viral epidemiology.

## 5. Conclusions

In this longitudinal tertiary-care laboratory archive, apparent age-specific viral detection patterns varied according to denominator choice, target availability, calendar period, and reconstructed testing configuration. HHV-6 accounted for the largest share of early-childhood viral-positive detections in the full archive; however, unequal target availability and very small complete-configuration pediatric positive denominators preclude interpreting this pattern as the epidemiology of HHV-6 CNS disease. VZV was the most frequently detected adult viral target in the full archive, although adult rankings varied by calendar period. Longitudinal CSF molecular studies should therefore report the observation unit, target availability, and denominator-specific measures explicitly and should distinguish laboratory molecular detection from clinically adjudicated infection.

## Figures and Tables

**Table 1:** Analytic structure by calendar period and reconstructed testing configuration.

Dataset Layer	Overall	Before 10 November 2020	On or after 10 November 2020	Selected/Non-14- Target Configurations	Reconstructed Complete 14-Target Configuration
Unique patients	4560	3271	1326	4095	506
Patient-date testing episodes	5353	3775	1578	4783	570
Target-level rows retained in the primary analysis	30,481	17,150	13,331	22,452	8029
Same-day patient–pathogen de-duplicated target results	30,334	17,067	13,267	22,354	7980
Positive target results	1058	846	212	1001	57
Viral-positive detections	1010	812	198	962	48

Data are presented as counts. Positive target results included bacterial, viral, and fungal targets; viral-positive detections were restricted to CMV, enterovirus, HSV-1, HSV-2, HHV-6, human parechovirus, and VZV. Calendar periods were defined as before versus on or after 10 November 2020. Configuration categories were assigned from the harmonized reporting-configuration field and verified against target representation within each patient-date episode; they denote reconstructed reporting configurations rather than definitive assay identities. Primary target-level counts retained same-day patient–pathogen duplicate rows. De-duplicated counts represent one result per patient, calendar date, and pathogen; the consolidation rule is described in Section 2.4. Patient counts are unique within each column and are not additive because 37 patients contributed records in both calendar periods and 41 contributed records in both configuration categories. CMV, cytomegalovirus; HHV-6, human herpesvirus 6; HSV-1, herpes simplex virus type 1; HSV-2, herpes simplex virus type 2; VZV, varicella-zoster virus.

## Data Availability

Individual-level laboratory information system data are not publicly available because they contain potentially re-identifiable clinical laboratory metadata and are subject to institutional data-governance and IRB restrictions. Aggregate data underlying the principal tables and figures, together with the analysis code and supporting documentation, are provided in the Supplementary Materials and Supplementary Data S1. Additional aggregate results are reported in Supplementary Tables S12–S17, and code for the corresponding descriptive analyses is included in Supplementary File S2. These materials permit verification of the reported aggregate point estimates and analysis procedures, but not independent reproduction of patient-cluster bootstrap intervals without the restricted patient-level analytic dataset. Requests for access to the restricted dataset will be considered subject to institutional data-governance and IRB approval.

## Supplementary Materials

The following supporting information can be downloaded at: https://www.mdpi.com/article/10.3390/pathogens15090905/s1, Table S1: Contextual pooled target-level positivity by age group; Table S2: Age-specific composition of CSF viral-positive detections, ranked by share within each age group; Table S3: Per-target positivity for the four most frequently detected herpesvirus targets by age group; Table S4: Exploratory deterministic threshold exercise for early-childhood HHV-6 ranking under hypothetical background detection; Table S5: Age-boundary sensitivity; Table S6: Longitudinal first-positive de-duplication sensitivity analysis; Table S7: Reconstructed configuration-specific summary; Table S8: Calendar-period sensitivity; Table S9: Sensitivity analysis excluding records obtained before 16 March 2010; Table S10: Documented assay, extraction-scope, and reconstructed-configuration characteristics and limits of retrospective reconstruction; Table S11: Cohort structure by age group; Table S12: Reconstructed source-data flow and result harmonization; Table S13: Testing architecture and viral-target availability by age group; Table S14: Viral-target availability by age group, calendar period, and reconstructed configuration; Table S15: Per-target positivity for all viral targets by age group; Table S16: Descriptive seasonality; Table S17: Same-day molecular co-detection; Supplementary Figure S1: Reconstructed source-to-analysis data flow; Supplementary Data S1: Aggregate source data underlying the principal tables and figures; Supplementary File S2: Reproducibility package containing analysis code, software requirements, and analysis documentation.
