# Supplementary material for: Denominator and Workflow Dependence of Age-Specific Viral Detection in Cerebrospinal Fluid
Source: Pathogens. 2026 Aug 27;15(9):905. doi: 10.3390/pathogens15090905 (PMC13610594; doi:10.3390/pathogens15090905)
Supplement: Supplementary file 1 [file pathogens-15-00905-s001.zip › Revised_Supplementary_Materials.pdf]

Supplementary Materials

Denominator and Workflow Dependence of Age-Specific Viral Detection in Cerebrospinal Fluid

Journal: Pathogens | Section: Viral Pathogens

Authors: Ji Yeon Kim, Hyeok-Jin Kwon, Jae-Sik Jeon, and Jae Kyung Kim

Corresponding author: Jae Kyung Kim, Department of Biomedical Laboratory Science, College of Health Sciences, Dankook University, Cheonan 31116, Republic of Korea; E-mail: nerowolf@naver.com

These supplementary materials provide numerical results underlying the main figures and sensitivity analyses and document extraction scope, testing architecture, all-virus denominators, seasonality, and molecular co-detection. Tables S1–S3 correspond to the principal denominator-specific measures; Table S4 is retained only as an exploratory mathematical exercise and is not used to support clinical attribution; Tables S5–S9 present sensitivity and contextual analyses; Table S10 documents assay, extraction-scope, and reconstructed-configuration information; Table S11 summarizes the cohort; Tables S12–S17 contain the source-data audit and additional descriptive analyses; and Supplementary Figure S1 shows the reconstructed source-to-analysis data flow.

Table S1: Contextual pooled target-level positivity by age group

Age group	Interpretable target-level results, n	Positive target-level results, n	Positivity, %	Patient-cluster bootstrap 95% CI
< 1 year	9,528	149	1.56	1.29–1.85
1–4 years	4,034	326	8.08	7.26–8.92
5–17 years	5,596	111	1.98	1.63–2.35
18–39 years	3,518	188	5.34	4.59–6.19
40–64 years	4,330	162	3.74	3.14–4.40
≥ 65 years	3,475	122	3.51	2.86–4.21

Percentile 95% confidence intervals were obtained from 10,000 patient-cluster bootstrap resamples. In each resample, patients were sampled with replacement and all of their target-level records were retained together. Contextual pooled target-level positivity was defined as the number of positive target-level CSF molecular results divided by all interpretable target results within each age group. It is a laboratory result-level measure shaped by target repertoire, panel width, and ordering practice and should not be interpreted as patient-, specimen-, or episode-level diagnostic yield. The bootstrap intervals quantify patient-resampling uncertainty conditional on the observed testing structure and do not incorporate structural ascertainment uncertainty.

Table S2: Age-specific composition of CSF viral-positive detections, ranked by share within each age group

Age group	Top pathogen	Second pathogen	Third pathogen	Total viral positives
< 1 year	HHV-6 91/120 (75.8%; 95% CI 66.1–85.1)	HSV-1 12/120 (10.0%; 95% CI 3.5–18.0)	VZV 8/120 (6.7%; 95% CI 2.6–11.7)	120
1–4 years	HHV-6 272/324 (84.0%; 95% CI 80.1–87.7)	HSV-1 33/324 (10.2%; 95% CI 7.1–13.5)	VZV 11/324 (3.4%; 95% CI 1.6–5.5)	324
5–17 years	VZV 44/108 (40.7%; 95% CI 31.2–50.4)	HHV-6 41/108 (38.0%; 95% CI 28.7–47.5)	HSV-1 20/108 (18.5%; 95% CI 11.4–26.3)	108
18–39 years	VZV 106/186 (57.0%; 95% CI 49.7–64.2)	HSV-1 48/186 (25.8%; 95% CI 19.6–32.1)	HSV-2 29/186 (15.6%; 95% CI 10.6–21.1)	186
40–64 years	VZV 73/157 (46.5%; 95% CI 38.9–54.3)	HSV-1 42/157 (26.8%; 95% CI 20.1–33.8)	HSV-2 35/157 (22.3%; 95% CI 16.0–29.1)	157
≥ 65 years	VZV 58/115 (50.4%; 95% CI 40.8–60.2)	HSV-1 36/115 (31.3%; 95% CI 22.2–40.6)	HSV-2 13/115 (11.3%; 95% CI 5.8–17.6)	115

Values are positive detections divided by all viral-positive detections within each age group. Only the three most frequent viral targets per age group are shown in the ranked table. Viral-positive composition is conditional on which targets were represented in testing and should not be interpreted as etiologic fractions of clinically adjudicated CNS infection. The documented historical herpesvirus repertoire included EBV, but the archived pre-September-2023 extraction query did not include an EBV-specific code; EBV was therefore outside the scope of the retained analytic dataset. Percentile 95% confidence intervals were obtained from 10,000 patient-cluster bootstrap resamples, with all records from each sampled patient retained together.

Table S3: Per-target positivity for the four most frequently detected herpesvirus targets by age group

Age group	HHV-6	HSV-1	HSV-2	VZV
< 1 year	91/1,219 (7.5%; 95% CI 5.9–9.1)	12/1,207 (1.0%; 95% CI 0.3–1.9)	4/1,206 (0.3%; 95% CI 0.0–0.9)	8/1,206 (0.7%; 95% CI 0.2–1.2)
1–4 years	272/854 (31.9%; 95% CI 28.6–35.2)	33/824 (4.0%; 95% CI 2.7–5.4)	2/823 (0.2%; 95% CI 0.0–0.6)	11/825 (1.3%; 95% CI 0.6–2.2)
5–17 years	41/1,050 (3.9%; 95% CI 2.8–5.1)	20/1,042 (1.9%; 95% CI 1.1–2.8)	0/1,042 (0.0%; bootstrap CI not reported*)	44/1,044 (4.2%; 95% CI 3.0–5.5)
18–39 years	2/585 (0.3%; 95% CI 0.0–0.9)	48/561 (8.6%; 95% CI 6.3–11.0)	29/562 (5.2%; 95% CI 3.4–7.0)	106/562 (18.9%; 95% CI 15.6–22.3)
40–64 years	4/672 (0.6%; 95% CI 0.1–1.2)	42/660 (6.4%; 95% CI 4.5–8.4)	35/660 (5.3%; 95% CI 3.6–7.1)	73/660 (11.1%; 95% CI 8.7–13.6)
≥ 65 years	4/508 (0.8%; 95% CI 0.0–2.2)	36/492 (7.3%; 95% CI 4.9–10.0)	13/491 (2.6%; 95% CI 1.3–4.2)	58/491 (11.8%; 95% CI 8.9–14.9)

Percentile 95% confidence intervals were obtained from 10,000 patient-cluster bootstrap resamples. Pseudonymized patients were sampled with replacement, and all target-level records belonging to each sampled patient were retained together. HHV-6, VZV, HSV-1, and HSV-2 were displayed in the main figure because they were identified descriptively after aggregation as the four most frequently detected herpesviruses; this was not a prespecified etiologic target set. Per-target denominators and positivity for all viral targets are provided in Table S15. *Because no HSV-2-positive events were observed in the 5–17-year group, the empirical patient-cluster bootstrap distribution was degenerate at 0, yielding a non-informative 95% confidence interval of 0.0–0.0; therefore, no confidence interval is reported.

Table S4: Exploratory deterministic threshold exercise for early-childhood HHV-6 ranking under hypothetical background detection

Age group	Observed HHV-6 detections, n	HHV-6 tests, n	Viral-positive detections, n	Second-ranked viral target, n	Observed HHV-6 share, %	Hypothetical test-level background-detection rate at which HHV-6 would no longer be the sole leading viral target, %
< 1 year	91	1,219	120	HSV-1, 12	75.8	6.5
1–4 years	272	854	324	HSV-1, 33	84.0	28.0

Values are retained as an exploratory mathematical exercise only. They do not estimate assay false-positive rates, iciHHV-6 prevalence, background HHV-6 detection, or individual causal attribution and are not used to validate the clinical significance of HHV-6 detection. For each age group, the threshold was calculated as (observed HHV-6 detections − detections of the second-ranked viral target) divided by the number of HHV-6 tests. At this threshold, the adjusted HHV-6 count would equal that of the second-ranked viral target. Thresholds were rounded to one decimal place.

Table S5: Age-boundary sensitivity of the headline findings

Scheme	Age cut-points	Highest positivity group	Highest HHV-6 share group
Scheme A	pediatric split 0/2/5/12/18/40/65	18–39 (5.3%)	0–1 years (265/318, 83.3%)
Scheme B	coarse 0/5/18/65	18–64 (4.5%)	0–4 years (363/444, 81.8%)
Scheme C	adult decades 0/1/5/18/30/40/50/60/70	1–4 (8.1%)	1–4 years (272/324, 84.0%)

The early-childhood HHV-6 detection ranking was consistent across the examined alternative age boundaries. This is a descriptive sensitivity result and does not constitute a formal equivalence or robustness test. The exact age group with the highest contextual pooled target-level positivity changed under some schemes. The listed cut-points indicate the lower bounds of successive age groups, with the final category extending from the last cut-point upward. Scheme C comprised < 1, 1–4, 5–17, 18–29, 30–39, 40–49, 50–59, 60–69, and ≥ 70 years.

Table S6: Longitudinal first-positive de-duplication sensitivity analysis

Age group	Target-level viral positives	First-positive viral detections	Leading target	Second target	Interpretation
< 1 year	120	111	HHV-6 87/111 (78.4%)	HSV-1 9/111 (8.1%)	HHV-6 remained leading
1–4 years	324	301	HHV-6 250/301 (83.1%)	HSV-1 33/301 (11.0%)	HHV-6 remained leading
5–17 years	108	105	HHV-6 and VZV, each 41/105 (39.0%)	HSV-1 20/105 (19.0%)	Balanced HHV-6/VZV
18–39 years	186	184	VZV 105/184 (57.1%)	HSV-1 47/184 (25.5%)	VZV remained leading
40–64 years	157	155	VZV 73/155 (47.1%)	HSV-1 41/155 (26.5%)	VZV remained leading
≥ 65 years	115	106	VZV 55/106 (51.9%)	HSV-1 33/106 (31.1%)	VZV remained leading

In the longitudinal first-positive de-duplication analysis, positive detections were sorted by pseudonymized patient identifier, pathogen, and date, and only the first positive result for each patient-pathogen combination was retained. Across all targets, 1,058 positive target-level results were reduced to 1,002 first patient-pathogen positive detections; viral positives were reduced from 1,010 to 962. Because the full testing denominator could not be de-duplicated consistently across the study period, this analysis evaluates only whether repeated positive detections materially changed positive-detection composition. Values should not be interpreted as patient-level positivity or incidence.

Table S7: Reconstructed configuration-specific summary

A. Testing-volume and same-day patient-pathogen de-duplication summary

Reconstructed testing configuration	Patient-date episodes	Raw target-level rows	Same-day patient-pathogen de-duplicated target results	Positive target results
**Selected/non-14-target configurations**	4,783	22,452	22,354	1,001
Reconstructed complete 14-target configuration	570	8,029	7,980	57
Total	5,353	30,481	30,334	1,058

Configuration categories were assigned from the harmonized reporting-configuration field and represent reconstructed testing configurations rather than definitive assay identities. Every episode assigned to the reconstructed complete 14-target configuration was verified to contain all 14 distinct panel targets after same-day patient–pathogen de-duplication. Same-day de-duplicated results were obtained by collapsing records for the same patient, date, and pathogen, with discordant groups classified as positive if any record was positive. This reduced 30,481 target-level rows to 30,334 results without changing the 1,058 positives. In the reconstructed complete 14-target configuration, the 49 excess raw rows reflected same-day patient–pathogen duplicates.

B. Reconstructed configuration-specific early-childhood viral composition

Age group	Selected/non-14-target configurations	Reconstructed complete 14-target configuration
< 1 year	HHV-6 led: 91/115 (79.1%)	Enterovirus led: 3/5 (60.0%); HHV-6 0/5
1–4 years	HHV-6 led: 268/313 (85.6%)	Enterovirus led: 6/11 (54.5%); HHV-6 4/11 (36.4%)

Denominators are viral-positive detections within each age group and reconstructed testing configuration. The complete 14-target estimates are exploratory/descriptive because only 5 and 11 viral-positive detections were available in infants and children aged 1–4 years, respectively; they are not used to infer epidemiological change or assay performance.

Table S8: Calendar-period sensitivity of headline age-related patterns

Metric	Before 10 November 2020	On or after 10 November 2020
Overall target-level summary	17,150 target-level results; 846 positive results (4.93%)	13,331 target-level results; 212 positive results (1.59%)
Highest contextual pooled target-level positivity group	18–39 years: 158/1,745 (9.05%)	1–4 years: 64/1,003 (6.38%)
Infants (< 1 year)	HHV-6: 78/100 (78.0%)	HHV-6: 13/20 (65.0%)
Children aged 1–4 years	HHV-6: 218/260 (83.8%)	HHV-6: 54/64 (84.4%)
Children aged 5–17 years	VZV led: 38/83 (45.8%)	HHV-6 led: 15/25 (60.0%)
Adult viral-positive pattern	VZV led ages 18–39 years (91/157, 58.0%), 40–64 years (67/135, 49.6%), and ≥ 65 years (43/77, 55.8%).	VZV led ages 18–39 years (15/29, 51.7%); HSV-1 led ages 40–64 years (7/22, 31.8%) and ≥ 65 years (21/38, 55.3%).
Interpretation	Early-childhood HHV-6 and adult VZV patterns were evident; VZV led at age 5–17 years.	Early-childhood HHV-6 persisted; HHV-6 led at age 5–17 years, and adult leading targets were less uniform.

Calendar-period strata were defined as before versus on or after 10 November 2020. These descriptive summaries are not assay-performance comparisons. Calendar period and workflow were not interchangeable because 1,008 of 1,578 later-period episodes remained in selected/non-14-target configurations. The split also coincided with the COVID-19 pandemic, so period differences may reflect pandemic-related changes in healthcare utilization, referral, pathogen circulation, and diagnostic behavior as well as assay availability. Target availability, ordering practices, and viral-positive denominators also differed across strata.

Table S9: Sensitivity analysis excluding records obtained before 16 March 2010

Age group	Full-dataset contextual pooled target-level positivity	After exclusion	Leading viral target after exclusion
< 1 year	149/9,528 (1.56%)	149/9,521 (1.56%)	HHV-6
1–4 years	326/4,034 (8.08%)	326/4,029 (8.09%)	HHV-6
5–17 years	111/5,596 (1.98%)	111/5,594 (1.98%)	VZV
18–39 years	188/3,518 (5.34%)	187/3,515 (5.32%)	VZV
40–64 years	162/4,330 (3.74%)	162/4,327 (3.74%)	VZV
≥ 65 years	122/3,475 (3.51%)	120/3,472 (3.46%)	VZV

The retained archive contained only 23 target-level records before 16 March 2010, including three positive CMV results; sustained record accumulation began thereafter. Exclusion of these records did not materially alter age-specific contextual pooled target-level positivity estimates or the leading viral target in any age group. Positivity values are positive target-level results divided by all interpretable target-level results within each age group; leading viral targets use the viral-positive denominator.

Table S10: Documented assay, extraction-scope, and reconstructed-configuration characteristics and limits of retrospective reconstruction

Characteristic	Before 10 November 2020	From 10 November 2020: selected/non-14-target reconstructed configurations	From 10 November 2020: reconstructed complete 14-target configuration
**Target(s) or target repertoire**	Seeplex Meningitis-V1 ACE Detection kit: HSV-1, HSV-2, VZV, EBV, CMV, and HHV-6. Seeplex Meningitis-B ACE Detection kit: *E. coli* K1, *H. influenzae*, *L. monocytogenes*, *N. meningitidis*, *S. agalactiae*, and *S. pneumoniae*.	Target-specific availability within continuing selected/non-14-target configurations could not be reconstructed consistently	*Escherichia coli* K1; *Haemophilus influenzae*; *Listeria monocytogenes*; *Neisseria meningitidis*; *Streptococcus agalactiae*; *Streptococcus pneumoniae*; CMV; enterovirus; HSV-1; HSV-2; HHV-6; human parechovirus; VZV; and *Cryptococcus neoformans/gattii*
**Assay or workflow**	Commercial conventional multiplex PCR kits with electrophoretic detection: Seeplex Meningitis-V1 ACE Detection and Seeplex Meningitis-B ACE Detection (Seegene Inc., Seoul, Republic of Korea).	Selected/non-14-target reporting configurations; definitive assay identifiers were not retained for all records	Reconstructed complete 14-target reporting configuration
**Ordering structure**	Clinically selected ordering through predefined herpesvirus, bacterial, combined, and other/non-standard target configurations.	Clinically selected testing	Reconstructed complete 14-target configuration (all 14 targets represented)
**Reported result unit**	Qualitative target-level result	Qualitative target-level result	One qualitative target-level result per target; 14 results per patient-date episode meeting the reconstructed complete 14-target definition
**Documentation status and limitations**	The commercial kit names, assay principle, and documented target repertoires were available. The archived pre-September-2023 extraction query explicitly enumerated retained test codes and did not include an EBV-specific code; EBV was therefore outside the scope of the retained analytic dataset despite being part of the documented historical herpesvirus repertoire. Historical EBV test volume/positivity and tests outside the archived query cannot be reconstructed from this dataset. Complete target-availability dates were also unavailable. In addition, CMV was retained in only 1.4–10.7% of pre-2020 selected episodes across age groups, demonstrating that target-specific retention within the archived extract was not uniform.	Selected testing continued after panel availability, but complete target-by-target availability dates and definitive assay identifiers were not retained. Categories therefore represent reconstructed reporting configurations.	Target repertoire and panel availability date were documented. The complete 14-target category was reconstructed from the presence of all 14 interpretable targets within a patient-date episode; the exact BioFire instrument model and assay generation were not recoverable for all records.

This table summarizes assay, extraction-scope, and workflow information recoverable from the archived laboratory files and author-confirmed documentation. Unavailable details were not inferred. The reconstructed categories should not be interpreted as definitive platform identities.

Table S11: Cohort structure by age group

Age group	Unique patients	Male, n (%)	Patient-date testing episodes	Age at testing, median (IQR), years	Target-level results	Positive target results	Positive patient-date episodes
**< 1 year**	1,332	781 (58.6)	1,518	0.12 (0.03–0.22)	9,528	149	147
**1–4 years**	801	457 (57.1)	882	2.34 (1.54–3.57)	4,034	326	319
**5–17 years**	950	606 (63.8)	1,106	10.20 (6.95–14.14)	5,596	111	110
**18–39 years**	522	252 (48.3)	609	29.51 (24.30–34.37)	3,518	188	186
**40–64 years**	576	321 (55.7)	701	53.75 (47.90–58.72)	4,330	162	156
**≥ 65 years**	433	226 (52.2)	537	75.03 (68.92–80.12)	3,475	122	121
**Overall**	4,560	2,608 (57.2)	5,353	6.95 (0.55–35.80)	30,481	1,058	1,039

Age was summarized at the patient-date testing-episode level. Sex distribution was calculated among unique patients within each age group; no missing or discordant patient-level sex records were identified. Fifty-two patients contributed testing episodes in more than one age group during the longitudinal study period, including two patients who contributed episodes in three age groups; age-group-specific patient counts are therefore not additive. A positive patient-date episode was defined as a patient-date group containing at least one positive target-level result and should not be interpreted as a unique specimen or clinically adjudicated episode. Positive target results included bacterial, viral, and fungal targets, whereas viral-positive detections were restricted to viral targets included in the analytic dataset.

Abbreviations: CMV, cytomegalovirus; EBV, Epstein–Barr virus; HHV-6, human herpesvirus 6; HSV-1, herpes simplex virus type 1; HSV-2, herpes simplex virus type 2; ME, meningitis/encephalitis; PCR, polymerase chain reaction; VZV, varicella-zoster virus.

Contents: Supplementary Tables S1–S17 and Supplementary Figure S1.

Supplementary Methods: Additional descriptive analyses

Testing architecture was summarized after same-day patient–pathogen de-duplication using the positive-wins rule for discordant duplicates. A target was considered represented in a patient-date episode when at least one interpretable result for that target was present. Configuration categories were assigned from the harmonized reporting-configuration field; every episode assigned to the reconstructed complete 14-target configuration was verified to contain all 14 distinct panel targets after same-day patient–pathogen de-duplication. Seasons were defined as winter (December–February), spring (March–May), summer (June–August), and autumn (September–November). Same-day co-detection required at least two distinct positive pathogen targets within one patient-date episode after patient–pathogen de-duplication. These additional analyses were descriptive; no new inferential hypothesis tests were introduced.

Table S12: Reconstructed source-data flow and result harmonization

Data-flow element	n	Definition / action
Archived source rows	31,249	26,853 through 31 Aug 2023 + 4,396 from 3 Sep 2023 through 30 Mar 2026
Retained: Negative	29,423	Mapped to negative
Retained: Positive	1,056	Mapped to positive
Retained: WeaklyPositive	1	Mapped to positive
Retained: Weak Positive	1	Mapped to positive
Excluded: blank result	699	Non-interpretable
Excluded: TEXT reference labels	48	47 “TEXT참조” + 1 “TEXT 참조” (original LIS labels: “see text”)
Excluded: Foot note reference labels	16	15 “Foot note 참조” + 1 “Foot note 참조” (original LIS labels: “see footnote”)
Excluded: n/N/p labels	5	n = 3; N = 1; p = 1
Excluded: missing or unusable age	0	All 30,481 interpretable rows had sufficient age information for age-group assignment

Final interpretable target-level rows	30,481	1,058 positive; 29,423 negative
Patient-date testing episodes	5,353	Same pseudonymized patient + calendar date
Reconstructed testing-configuration classification	5,353	4,783 selected/non-14-target episodes; 570 reconstructed complete 14-target episodes
Unique patients	4,560	Pseudonymized patient identifier

The archived source extraction files permitted reconstruction of the result-label flow shown above. The Korean strings are retained verbatim from the original laboratory information system (LIS): “TEXT참조”/“TEXT 참조” means “see text,” and “Foot note 참조”/“Foot note 참조” means “see footnote.” The retained archived extracts contained no rows dated 1–2 September 2023; because no independent source log was available, this audit cannot distinguish an absence of testing from noncoverage by the retained extracts on those two dates. The archived materials documented a rule-based result mapping; no separate case-by-case manual recoding step was documented or identified in the materials available for re-audit. This audit does not establish completeness for tests that were outside the archived extraction queries. No additional interpretable rows were excluded for missing age. The additional descriptive analyses used the documented result mapping in the analysis-ready master dataset.

Supplementary Figure S1: Reconstructed source-to-analysis data flow

Archived laboratory source extracts
31,249 target-level rows

↓

Excluded non-interpretable/reference result labels
768 rows

↓

Interpretable harmonized target-level results
30,481 rows (1,058 positive; 29,423 negative)

↓

Patient-date testing episodes
5,353 episodes

↓

Reconstructed testing-configuration classification
4,783 selected/non-14-target episodes
570 reconstructed complete 14-target episodes

↓

Final analytic cohort
30,481 target-level results; 5,353 episodes; 4,560 unique patients

Table S13: Testing architecture and viral-target availability by age group

A. Episode-level testing intensity. Complete 14-target indicates the reconstructed complete 14-target reporting configuration.

Age group	Unique patients	Patient-date episodes	Distinct viral targets/episode, median (IQR)	Complete 14-target n (%)
< 1 year	1332	1518	4 (4–4)	219 (14.4%)
1–4 years	801	882	4 (4–4)	34 (3.9%)
5–17 years	950	1106	4 (4–4)	58 (5.2%)

18–39 years	522	609	4 (4–4)	68 (11.2%)
40–64 years	576	701	4 (4–4)	100 (14.3%)
≥ 65 years	433	537	4 (4–4)	91 (16.9%)

B. Episode-level availability of the four most frequently detected herpesvirus targets.

Age group	HHV-6 tested n (%)	VZV tested n (%)	HSV-1 tested n (%)	HSV-2 tested n (%)
< 1 year	1213 (79.9%)	1202 (79.2%)	1201 (79.1%)	1202 (79.2%)
1–4 years	848 (96.1%)	820 (93.0%)	820 (93.0%)	820 (93.0%)
5–17 years	1043 (94.3%)	1036 (93.7%)	1036 (93.7%)	1036 (93.7%)
18–39 years	583 (95.7%)	559 (91.8%)	559 (91.8%)	559 (91.8%)
40–64 years	667 (95.1%)	656 (93.6%)	656 (93.6%)	656 (93.6%)
≥ 65 years	506 (94.2%)	489 (91.1%)	489 (91.1%)	489 (91.1%)

C. Episode-level availability of the remaining viral targets. Percentages use all patient-date testing episodes within each age group as the denominator. Target representation was assessed after same-day patient–pathogen de-duplication. Availability denotes retained-and-reported target representation in the analytic archive and should not be interpreted as complete institutional testing volume. In the retained archive, enterovirus and parechovirus were represented only in episodes meeting the reconstructed complete 14-target definition; accordingly, their age-specific availability percentages are numerically identical to the corresponding proportions of reconstructed complete 14-target episodes.

Age group	CMV tested n (%)	Enterovirus tested n (%)	Parechovirus tested n (%)
< 1 year	256 (16.9%)	219 (14.4%)	219 (14.4%)
1–4 years	46 (5.2%)	34 (3.9%)	34 (3.9%)
5–17 years	70 (6.3%)	58 (5.2%)	58 (5.2%)
18–39 years	100 (16.4%)	68 (11.2%)	68 (11.2%)
40–64 years	138 (19.7%)	100 (14.3%)	100 (14.3%)
≥ 65 years	122 (22.7%)	91 (16.9%)	91 (16.9%)

Table S14: Viral-target availability by age group, calendar period, and reconstructed configuration

A. Herpesvirus target availability. No reconstructed complete 14-target episodes occurred before 10 November 2020.

Age	Calendar period	Configuration	Episodes	HHV-6 n (%)	VZV n (%)	HSV-1 n (%)	HSV-2 n (%)
< 1 year	Pre-10 Nov 2020	Selected/non-14	1147	861 (75.1%)	850 (74.1%)	849 (74.0%)	850 (74.1%)
< 1 year	On/after 10 Nov 2020	Selected/non-14	152	133 (87.5%)	133 (87.5%)	133 (87.5%)	133 (87.5%)
< 1 year	On/after 10 Nov 2020	Complete 14-target	219	219 (100.0%)	219 (100.0%)	219 (100.0%)	219 (100.0%)
1–4 years	Pre-10 Nov 2020	Selected/non-14	722	688 (95.3%)	660 (91.4%)	660 (91.4%)	660 (91.4%)
1–4 years	On/after 10 Nov 2020	Selected/non-14	126	126 (100.0%)	126 (100.0%)	126 (100.0%)	126 (100.0%)
1–4 years	On/after 10 Nov 2020	Complete 14-target	34	34 (100.0%)	34 (100.0%)	34 (100.0%)	34 (100.0%)
5–17 years	Pre-10 Nov 2020	Selected/non-14	787	726 (92.2%)	719 (91.4%)	719 (91.4%)	719 (91.4%)
5–17 years	On/after 10 Nov 2020	Selected/non-14	261	259 (99.2%)	259 (99.2%)	259 (99.2%)	259 (99.2%)
5–17 years	On/after 10 Nov 2020	Complete 14-target	58	58 (100.0%)	58 (100.0%)	58 (100.0%)	58 (100.0%)
18–39 years	Pre-10 Nov 2020	Selected/non-14	421	396 (94.1%)	372 (88.4%)	372 (88.4%)	372 (88.4%)
18–39 years	On/after 10 Nov 2020	Selected/non-14	120	119 (99.2%)	119 (99.2%)	119 (99.2%)	119 (99.2%)
18–39 years	On/after 10 Nov 2020	Complete 14-target	68	68 (100.0%)	68 (100.0%)	68 (100.0%)	68 (100.0%)
40–64 years	Pre-10 Nov 2020	Selected/non-14	418	386 (92.3%)	375 (89.7%)	375 (89.7%)	375 (89.7%)
40–64 years	On/after 10 Nov 2020	Selected/non-14	183	181 (98.9%)	181 (98.9%)	181 (98.9%)	181 (98.9%)
40–64 years	On/after 10 Nov 2020	Complete 14-target	100	100 (100.0%)	100 (100.0%)	100 (100.0%)	100 (100.0%)

≥ 65 years	Pre-10 Nov 2020	Selected/non-14	280	256 (91.4%)	239 (85.4%)	239 (85.4%)	239 (85.4%)
≥ 65 years	On/after 10 Nov 2020	Selected/non-14	166	159 (95.8%)	159 (95.8%)	159 (95.8%)	159 (95.8%)
≥ 65 years	On/after 10 Nov 2020	Complete 14-target	91	91 (100.0%)	91 (100.0%)	91 (100.0%)	91 (100.0%)

B. Other viral-target availability. Percentages use patient-date episodes within each age × calendar-period × configuration stratum as the denominator. The table shows the selection-by-testing structure directly; it is not an assay-performance comparison. The very low pre-2020 CMV representation despite its inclusion in the documented herpesvirus kit repertoire demonstrates target-specific incompleteness of the retained archive.

Age	Calendar period	Configuration	Episodes	CMV n (%)	Enterovirus n (%)	Parechovirus n (%)
< 1 year	Pre-10 Nov 2020	Selected/non-14	1147	32 (2.8%)	0 (0.0%)	0 (0.0%)
< 1 year	On/after 10 Nov 2020	Selected/non-14	152	5 (3.3%)	0 (0.0%)	0 (0.0%)
< 1 year	On/after 10 Nov 2020	Complete 14-target	219	219 (100.0%)	219 (100.0%)	219 (100.0%)
1–4 years	Pre-10 Nov 2020	Selected/non-14	722	12 (1.7%)	0 (0.0%)	0 (0.0%)
1–4 years	On/after 10 Nov 2020	Selected/non-14	126	0 (0.0%)	0 (0.0%)	0 (0.0%)
1–4 years	On/after 10 Nov 2020	Complete 14-target	34	34 (100.0%)	34 (100.0%)	34 (100.0%)
5–17 years	Pre-10 Nov 2020	Selected/non-14	787	11 (1.4%)	0 (0.0%)	0 (0.0%)
5–17 years	On/after 10 Nov 2020	Selected/non-14	261	1 (0.4%)	0 (0.0%)	0 (0.0%)
5–17 years	On/after 10 Nov 2020	Complete 14-target	58	58 (100.0%)	58 (100.0%)	58 (100.0%)
18–39 years	Pre-10 Nov 2020	Selected/non-14	421	32 (7.6%)	0 (0.0%)	0 (0.0%)
18–39 years	On/after 10 Nov 2020	Selected/non-14	120	0 (0.0%)	0 (0.0%)	0 (0.0%)
18–39 years	On/after 10 Nov 2020	Complete 14-target	68	68 (100.0%)	68 (100.0%)	68 (100.0%)
40–64 years	Pre-10 Nov 2020	Selected/non-14	418	38 (9.1%)	0 (0.0%)	0 (0.0%)
40–64 years	On/after 10 Nov 2020	Selected/non-14	183	0 (0.0%)	0 (0.0%)	0 (0.0%)
40–64 years	On/after 10 Nov 2020	Complete 14-target	100	100 (100.0%)	100 (100.0%)	100 (100.0%)
≥ 65 years	Pre-10 Nov 2020	Selected/non-14	280	30 (10.7%)	0 (0.0%)	0 (0.0%)
≥ 65 years	On/after 10 Nov 2020	Selected/non-14	166	1 (0.6%)	0 (0.0%)	0 (0.0%)
≥ 65 years	On/after 10 Nov 2020	Complete 14-target	91	91 (100.0%)	91 (100.0%)	91 (100.0%)

Table S15: Per-target positivity for all viral targets by age group

Age group	Viral target	Interpretable target-level results, n	Positive, n	Target-level positivity, %
< 1 year	CMV	256	1	0.39
< 1 year	Enterovirus	219	3	1.37
< 1 year	HSV-1	1207	12	0.99
< 1 year	HSV-2	1206	4	0.33
< 1 year	HHV-6	1219	91	7.47
< 1 year	Parechovirus	219	1	0.46
< 1 year	VZV	1206	8	0.66
1–4 years	CMV	46	0	0.00
1–4 years	Enterovirus	34	6	17.65
1–4 years	HSV-1	824	33	4.00
1–4 years	HSV-2	823	2	0.24
1–4 years	HHV-6	854	272	31.85
1–4 years	Parechovirus	34	0	0.00
1–4 years	VZV	825	11	1.33

5–17 years	CMV	70	0	0.00
5–17 years	Enterovirus	58	3	5.17
5–17 years	HSV-1	1042	20	1.92
5–17 years	HSV-2	1042	0	0.00
5–17 years	HHV-6	1050	41	3.90
5–17 years	Parechovirus	58	0	0.00
5–17 years	VZV	1044	44	4.21
18–39 years	CMV	100	1	1.00
18–39 years	Enterovirus	68	0	0.00
18–39 years	HSV-1	561	48	8.56
18–39 years	HSV-2	562	29	5.16
18–39 years	HHV-6	585	2	0.34
18–39 years	Parechovirus	68	0	0.00
18–39 years	VZV	562	106	18.86
40–64 years	CMV	140	3	2.14
40–64 years	Enterovirus	102	0	0.00
40–64 years	HSV-1	660	42	6.36
40–64 years	HSV-2	660	35	5.30
40–64 years	HHV-6	672	4	0.60
40–64 years	Parechovirus	102	0	0.00
40–64 years	VZV	660	73	11.06
≥ 65 years	CMV	122	4	3.28
≥ 65 years	Enterovirus	91	0	0.00
≥ 65 years	HSV-1	492	36	7.32
≥ 65 years	HSV-2	491	13	2.65
≥ 65 years	HHV-6	508	4	0.79
≥ 65 years	Parechovirus	91	0	0.00
≥ 65 years	VZV	491	58	11.81

Per-target positivity uses the primary target-level denominator and therefore retains same-day duplicate rows, consistent with the main per-target analysis. The four herpesviruses displayed in Figure 1 were selected descriptively because they accounted for 988/1,010 viral detections; all seven viral targets are shown here.

Table S16: Descriptive seasonality

A. Distribution of patient-date testing episodes by season within each age group.

Age group	Winter n (%)	Spring n (%)	Summer n (%)	Autumn n (%)
< 1 year	337 (22.2%)	348 (22.9%)	480 (31.6%)	353 (23.3%)
1–4 years	149 (16.9%)	237 (26.9%)	297 (33.7%)	199 (22.6%)
5–17 years	180 (16.3%)	279 (25.2%)	402 (36.3%)	245 (22.2%)
18–39 years	122 (20.0%)	172 (28.2%)	190 (31.2%)	125 (20.5%)
40–64 years	175 (25.0%)	187 (26.7%)	173 (24.7%)	166 (23.7%)
≥ 65 years	132 (24.6%)	139 (25.9%)	136 (25.3%)	130 (24.2%)

B. Descriptive viral target-level positivity by season. Seasons were defined as winter (December–February), spring (March–May), summer (June–August), and autumn (September–November). These unadjusted summaries are contextual only and do not separate season from calendar period, workflow, or target availability. Enterovirus and parechovirus target results in the retained archive occurred only on or after 10 November 2020 in reconstructed complete 14-target configurations; their seasonal distributions therefore remain confounded by calendar period and configuration availability.

Season	Viral target	Target-level results, n	Positive, n	Target-level positivity, %
Winter	CMV	178	3	1.69
Winter	Enterovirus	139	0	0.00
Winter	HSV-1	960	37	3.85
Winter	HSV-2	959	21	2.19
Winter	HHV-6	989	78	7.89
Winter	Parechovirus	139	0	0.00
Winter	VZV	959	62	6.47
Spring	CMV	130	4	3.08
Spring	Enterovirus	104	0	0.00
Spring	HSV-1	1251	73	5.84
Spring	HSV-2	1252	21	1.68
Spring	HHV-6	1282	122	9.52
Spring	Parechovirus	104	0	0.00
Spring	VZV	1251	76	6.08
Summer	CMV	197	2	1.02
Summer	Enterovirus	156	7	4.49
Summer	HSV-1	1497	46	3.07
Summer	HSV-2	1495	21	1.40
Summer	HHV-6	1518	132	8.70
Summer	Parechovirus	156	1	0.64
Summer	VZV	1499	91	6.07
Autumn	CMV	229	0	0.00
Autumn	Enterovirus	173	5	2.89
Autumn	HSV-1	1078	35	3.25
Autumn	HSV-2	1078	20	1.86
Autumn	HHV-6	1099	82	7.46
Autumn	Parechovirus	173	0	0.00
Autumn	VZV	1079	71	6.58

Table S17: Same-day molecular co-detection

Measure	n	Definition / context
Patient-date episodes with ≥1 positive target	1039	Denominator for positive episodes
Episodes with ≥2 distinct positive targets	18	1.7% of positive patient-date episodes
Viral–viral co-detection	17	Distinct viral targets detected on same patient-date
Viral–nonviral co-detection	1	At least one viral and one nonviral target
HHV-6-positive patient-date episodes	414	At least one HHV-6-positive target result

HHV-6-positive episodes with another positive target	9	2.2% of HHV-6-positive patient-date episodes

**Positive-target combination**	**Patient-date episodes, n**
HSV-1 + VZV	7
HHV-6 + VZV	5
HHV-6 + HSV-1	4
HSV-1 + HSV-2 + VZV	1
S. agalactiae + VZV	1

Co-detection was defined after same-day patient–pathogen de-duplication. These are molecular co-detections and should not be interpreted as clinically adjudicated coinfections. No episode contained multiple distinct nonviral targets without a viral target.
